# The use of Qualitative Comparative Analysis (QCA) to address causality in complex systems: a systematic review of research on public health interventions

**DOI:** 10.1186/s12889-021-10926-2

**Published:** 2021-05-07

**Authors:** Benjamin Hanckel, Mark Petticrew, James Thomas, Judith Green

**Affiliations:** 1 Institute for Culture and Society, Western Sydney University, Sydney, Australia; 2 Department of Public Health, Environments and Society, LSHTM, London, UK; 3 UCL Institute of Education, University College London, London, UK; 4 Wellcome Centre for Cultures & Environments of Health, University of Exeter, Exeter, UK

**Keywords:** Complexity, Context, Evaluation, Public health, Intervention, Qualitative Comparative Analysis, Systematic review

## Abstract

**Background:**

Qualitative Comparative Analysis (QCA) is a method for identifying the configurations of conditions that lead to specific outcomes. Given its potential for providing evidence of causality in complex systems, QCA is increasingly used in evaluative research to examine the uptake or impacts of public health interventions. We map this emerging field, assessing the strengths and weaknesses of QCA approaches identified in published studies, and identify implications for future research and reporting.

**Methods:**

PubMed, Scopus and Web of Science were systematically searched for peer-reviewed studies published in English up to December 2019 that had used QCA methods to identify the conditions associated with the uptake and/or effectiveness of interventions for public health. Data relating to the interventions studied (settings/level of intervention/populations), methods (type of QCA, case level, source of data, other methods used) and reported strengths and weaknesses of QCA were extracted and synthesised narratively.

**Results:**

The search identified 1384 papers, of which 27 (describing 26 studies) met the inclusion criteria. Interventions evaluated ranged across: nutrition/obesity (*n* = 8); physical activity (*n* = 4); health inequalities (*n* = 3); mental health (*n* = 2); community engagement (*n* = 3); chronic condition management (*n* = 3); vaccine adoption or implementation (*n* = 2); programme implementation (*n *= 3); breastfeeding (*n *= 2), and general population health (*n* = 1). The majority of studies (*n* = 24) were of interventions solely or predominantly in high income countries. Key strengths reported were that QCA provides a method for addressing causal complexity; and that it provides a systematic approach for understanding the mechanisms at work in implementation across contexts. Weaknesses reported related to data availability limitations, especially on ineffective interventions. The majority of papers demonstrated good knowledge of cases, and justification of case selection, but other criteria of methodological quality were less comprehensively met.

**Conclusion:**

QCA is a promising approach for addressing the role of context in complex interventions, and for identifying causal configurations of conditions that predict implementation and/or outcomes when there is sufficiently detailed understanding of a series of comparable cases. As the use of QCA in evaluative health research increases, there may be a need to develop advice for public health researchers and journals on minimum criteria for quality and reporting.

**Supplementary Information:**

The online version contains supplementary material available at 10.1186/s12889-021-10926-2.

## Background

Interest in the use of Qualitative Comparative Analysis (QCA) arises in part from growing recognition of the need to broaden methodological capacity to address causality in complex systems [1–3]. Guidance for researchers for evaluating complex interventions suggests process evaluations [4, 5] can provide evidence on the mechanisms of change, and the ways in which context affects outcomes. However, this does not address the more fundamental problems with trial and quasi-experimental designs arising from system complexity [6]. As Byrne notes, the key characteristic of complex systems is ‘emergence’ [7]: that is, effects may accrue from combinations of components, in contingent ways, which cannot be reduced to any one level. Asking about ‘what works’ in complex systems is not to ask a simple question about whether an intervention has particular effects, but rather to ask: “how the intervention works in relation to all existing components of the system and to other systems and their sub-systems that intersect with the system of interest” [7]. Public health interventions are typically attempts to effect change in systems that are themselves dynamic; approaches to evaluation are needed that can deal with emergence [8]. In short, understanding the uptake and impact of interventions requires methods that can account for the complex interplay of intervention conditions and system contexts.

To build a useful evidence base for public health, evaluations thus need to assess not just whether a particular intervention (or component) causes specific change in one variable, in controlled circumstances, but whether those interventions shift systems, and how specific conditions of interventions and setting contexts interact to lead to anticipated outcomes. There have been a number of calls for the development of methods in intervention research to address these issues of complex causation [9–11], including calls for the greater use of case studies to provide evidence on the important elements of context [12, 13]. One approach for addressing causality in complex systems is Qualitative Comparative Analysis (QCA): a systematic way of comparing the outcomes of different combinations of system components and elements of context (‘conditions’) across a series of cases.

### The potential of qualitative comparative analysis

QCA is an approach developed by Charles Ragin [14, 15], originating in comparative politics and macrosociology to address questions of comparative historical development. Using set theory, QCA methods explore the relationships between ‘conditions’ and ‘outcomes’ by identifying configurations of necessary and sufficient conditions for an outcome. The underlying logic is different from probabilistic reasoning, as the causal relationships identified are not inferred from the (statistical) likelihood of them being found by chance, but rather from comparing sets of conditions and their relationship to outcomes. It is thus more akin to the generative conceptualisations of causality in realist evaluation approaches [16]. QCA is a non-additive and non-linear method that emphasises diversity, acknowledging that different paths can lead to the same outcome. For evaluative research in complex systems [17], QCA therefore offers a number of benefits, including: that QCA can identify more than one causal pathway to an outcome (equifinality); that it accounts for conjectural causation (where the presence or absence of conditions in relation to other conditions might be key); and that it is asymmetric with respect to the success or failure of outcomes. That is, that specific factors explain success does not imply that their absence leads to failure (causal asymmetry).

QCA was designed, and is typically used, to compare data from a medium N (10–50) series of cases that include those with and those without the (dichotomised) outcome. Conditions can be dichotomised in ‘crisp sets’ (csQCA) or represented in ‘fuzzy sets’ (fsQCA), where set membership is calibrated (either continuously or with cut offs) between two extremes representing fully in (1) or fully out (0) of the set. A third version, multi-value QCA (mvQCA), infrequently used, represents conditions as ‘multi-value sets’, with multinomial membership [18]. In calibrating set membership, the researcher specifies the critical qualitative anchors that capture differences in kind (full membership and full non-membership), as well as differences in degree in fuzzy sets (partial membership) [15, 19]. Data on outcomes and conditions can come from primary or secondary qualitative and/or quantitative sources. Once data are assembled and coded, truth tables are constructed which “list the logically possible combinations of causal conditions” [15], collating the number of cases where those configurations occur to see if they share the same outcome. Analysis of these truth tables assesses first whether any conditions are individually necessary or sufficient to predict the outcome, and then whether any configurations of conditions are necessary or sufficient. Necessary conditions are assessed by examining causal conditions shared by cases with the same outcome, whilst identifying sufficient conditions (or combinations of conditions) requires examining cases with the same causal conditions to identify if they have the same outcome [15]. However, as Legewie argues, the presence of a condition, or a combination of conditions in actual datasets, are likely to be “‘quasi-necessary’ or ‘quasi-sufficient’ in that the causal relation holds in a great majority of cases, but some cases deviate from this pattern” [20]. Following reduction of the complexity of the model, the final model is tested for coverage (the degree to which a configuration accounts for instances of an outcome in the empirical cases; the proportion of cases belonging to a particular configuration) and consistency (the degree to which the cases sharing a combination of conditions align with a proposed subset relation). The result is an analysis of complex causation, “defined as a situation in which an outcome may follow from several different combinations of causal conditions” [15] illuminating the ‘causal recipes’, the causally relevant conditions or configuration of conditions that produce the outcome of interest.

QCA, then, has promise for addressing questions of complex causation, and recent calls for the greater use of QCA methods have come from a range of fields related to public health, including health research [17], studies of social interventions [7], and policy evaluation [21, 22]. In making arguments for the use of QCA across these fields, researchers have also indicated some of the considerations that must be taken into account to ensure robust and credible analyses. There is a need, for instance, to ensure that ‘contradictions’, where cases with the same configurations show different outcomes, are resolved and reported [15, 23, 24]. Additionally, researchers must consider the ratio of cases to conditions, and limit the number of conditions to cases to ensure the validity of models [25]. Marx and Dusa, examining crisp set QCA, have provided some guidance to the ‘ceiling’ number of conditions which can be included relative to the number of cases to increase the probability of models being valid (that is, with a low probability of being generated through random data) [26].

There is now a growing body of published research in public health and related fields drawing on QCA methods. This is therefore a timely point to map the field and assess the potential of QCA as a method for contributing to the evidence base for what works in improving public health. To inform future methodological development of robust methods for addressing complexity in the evaluation of public health interventions, we undertook a systematic review to map existing evidence, identify gaps in, and strengths and weakness of, the QCA literature to date, and identify the implications of these for conducting and reporting future QCA studies for public health evaluation. We aimed to address the following specific questions [27]:

1. How is QCA used for public health evaluation? What populations, settings, methods used in source case studies, unit/s and level of analysis (‘cases’), and ‘conditions’ have been included in QCA studies?

2. What strengths and weaknesses have been identified by researchers who have used QCA to understand complex causation in public health evaluation research?

3. What are the existing gaps in, and strengths and weakness of, the QCA literature in public health evaluation, and what implications do these have for future research and reporting of QCA studies for public health?

## Methods

This systematic review was registered with the International Prospective Register of Systematic Reviews (PROSPERO) on 29 April 2019 (CRD42019131910). A protocol was prepared in accordance with the Preferred Reporting Items for Systematic Reviews and Meta-Analysis Protocols (PRISMA-P) 2015 statement [28], and published in 2019 [27], where the methods are explained in detail. EPPI-Reviewer 4 was used to manage the process and undertake screening of abstracts [29].

### Search strategy

We searched for peer-reviewed published papers in English, which used QCA methods to examine causal complexity in evaluating the implementation, uptake and/or effects of a public health intervention, in any region of the world, for any population. ‘Public health interventions’ were defined as those which aim to promote or protect health, or prevent ill health, in the population. No date exclusions were made, and papers published up to December 2019 were included.

Search strategies used the following phrases “Qualitative Comparative Analysis” and “QCA”, which were combined with the keywords “health”, “public health”, “intervention”, and “wellbeing”. See Additional file 1 for an example. Searches were undertaken on the following databases: PubMed, Web of Science, and Scopus. Additional searches were undertaken on Microsoft Academic and Google Scholar in December 2019, where the first pages of results were checked for studies that may have been missed in the initial search. No additional studies were identified. The list of included studies was sent to experts in QCA methods in health and related fields, including authors of included studies and/or those who had published on QCA methodology. This generated no additional studies within scope, but a suggestion to check the COMPASSS (Comparative Methods for Systematic Cross-Case Analysis) database; this was searched, identifying one further study that met the inclusion criteria [30]. COMPASSS (https://compasss.org/) collates publications of studies using comparative case analysis.

We excluded studies where no intervention was evaluated, which included studies that used QCA to examine public health infrastructure (i.e. staff training) without a specific health outcome, and papers that report on prevalence of health issues (i.e. prevalence of child mortality). We also excluded studies of health systems or services interventions where there was no public health outcome.

### Selection

**Fig. 1:**
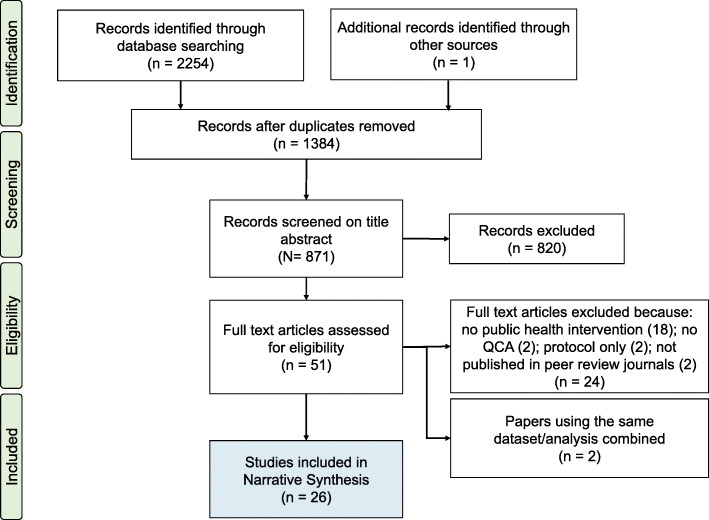
Flow Diagram

After retrieval, and removal of duplicates, titles and abstracts were screened by one of two authors (BH or JG). Double screening of all records was assisted by EPPI Reviewer 4’s machine learning function. Of the 1384 papers identified after duplicates were removed, we excluded 820 after review of titles and abstracts (Fig. 1). The excluded studies included: a large number of papers relating to ‘quantitative coronary angioplasty’ and some which referred to the Queensland Criminal Code (both of which are also abbreviated to ‘QCA’); papers that reported methodological issues but not empirical studies; protocols; and papers that used the phrase ‘qualitative comparative analysis’ to refer to qualitative studies that compared different sub-populations or cases within the study, but did not include formal QCA methods.

Full texts of the 51 remaining studies were screened by BH and JG for inclusion, with 10 papers double coded by both authors, with complete agreement. Uncertain inclusions were checked by the third author (MP). Of the full texts, 24 were excluded because: they did not report a public health intervention (*n* = 18); had used a methodology inspired by QCA, but had not undertaken a QCA (*n* = 2); were protocols or methodological papers only (*n* = 2); or were not published in peer-reviewed journals (*n* = 2) (see Fig. 1).

Data were extracted manually from the 27 remaining full texts by BH and JG. Two papers relating to the same research question and dataset were combined, such that analysis was by study (*n* = 26) not by paper. We retrieved data relating to: publication (journal, first author country affiliation, funding reported); the study setting (country/region setting, population targeted by the intervention(s)); intervention(s) studied; methods (aims, rationale for using QCA, crisp or fuzzy set QCA, other analysis methods used); data sources drawn on for cases (source [primary data, secondary data, published analyses], qualitative/quantitative data, level of analysis, number of cases, final causal conditions included in the analysis); outcome explained; and claims made about strengths and weaknesses of using QCA (see Table 1). Data were synthesised narratively, using thematic synthesis methods [31, 32], with interventions categorised by public health domain and level of intervention.

**Table 1:** Studies included in review: summary of data extracted

Publication details		Study details extracted											Indicators of reporting quality						
Lead Author	Publication date	Public health domain	Rationale for using QCA	Crisp or Fuzzy Set?	Country/region setting	Population	Intervention evaluated	Data used for analysis - source	Type of data (qual/qant)	Case level of analysis	Number of cases/potential cases	Outcome (s) (phenomena explained)	Evidence of familiarity of cases	Justification for selection of cases	Calibration of set membership scores discussed in detail and justified	Raw data matrix available	Truth tables reported	Solution formula reported and justified	Consistency and coverage measures reported
Bianchi	2018a	Nutrition /obesity	“to identify configurations of intervention characteristics associated with, and those not found to be associated with, statistically significant reductions in the demand for meat”	Crisp	Any (included studies all high income countries)	Any	Micro-environment interventions to reduce meat consumption	Published analyses (systematic review)	Quant	Intervention component	21 out of total of 22 interventions identified	Reduction in consumption, purchase, or selection of meat	Y	Y	N	N	N	Y	Y
Bianchi	2018b	Nutrition/obesity	“to identify combinations of intervention characteristics associated with significant reductions in the demand for meat”	Crisp	Any	Any	Interventions targeting conscious determinants of human behaviour to reduce the demand for meat.	Published analyses (systematic review)	Quant	Intervention component	31 out of a total of 59 interventions identified	Reductions in actual or intended meat consumption	Y	Y	N	N	N	N	Y
Blackman	2011	Health inequalities	“to find configurations of these conditions with either the narrowing or not narrowing outcomes.”; “enables causal arguments to be made rigorously by creating a very close correspondence between theory and data analysis, analysing evidence in ways that directly address theoretical arguments about what matters to achieve some outcome: how it happens. This makes QCA especially appropriate for policy research; the process of defining conditions is then also a process of identifying conditions necessary for a policy outcome.”	Crisp	UK	Those living in ‘deprived’ areas/regions, and at-risk of premature death from cancer and cardiovascular disease (CVD)	Target setting to reduce inequalities (Local policies and service delivery to identified disadvantaged areas)	Primary data (generated for study) Secondary data	Quant	Region/ subnational area - England’s Spearhead areas	27 of possible 70 (27 complete questionairres returned for both cancer and CVD)	Not/narrowing cancer gap; or not/narrowing CVD gap	Y	Y	N	N	Y	Y	N
Blackman	2010	Health inequalities	“QCA […] assumes that outcomes are produced by variables acting together in combination, including the possibility of different combinations leading to the same outcome”; “using fsQCA to explore how the attributes configured against each outcome.”	Crisp	UK - North-West England	Those living in ‘deprived’ areas/regions, and at-risk of premature death from cancer and circulatory diseases	Local policies and service delivery to identified disadvantaged areas	Primary data (generated for study) Secondary data	Qual and Quant	Region/ subnational area - local strategic partnerships (LSP)	15 local authority areas in North West England of 21 LSP areas in the North West region that were among the most deprived 88 local authority areas in England (14 and 15 complete questionairries were returned for circulatory diseases and cancers, respectively)	Narrowing OR widening cancer gap; or narrowing OR widening circulatory diseases gap	Y	Y	N	N	Y	Y	N
Blackman	2013	Health inequalities	“enabling causal pathways to be discerned from how sets of conditions combine with particular outcomes: in this instance, whether inequalities in conception rates do or do not narrow, compared with the England average.”	Crisp	England, deprived local authority areas	Women under 18	Local policies and service delivery addressing pregnancy in those aged under-18	Primary data (generated for study) Secondary data	Quant	Region/ subnational area – local authority	27 from 70 local authority areas	A narrowing teenage pregnancy rate gap compared to the national average OR a gap that had not been narrowing.	Y	Y	Y	N	Y	Y	Y
Bruer	2018	Mental health	Goal to “to evaluate outcomes and processes simultaneously … [QCA] holds promise as a way to do this”	Fuzzy	Nepal, West Chitwan	Primary care patients	Programme for Improving Mental Healthcare’ (PRIME) Mental Health Care Plans (MHCPs)	Secondary data	Quant	Organisation - health facilities	10 of the 10 health facilities in Chitwan where the PRIME MHCPs were implemented	Effectiveness of the intervention:increased service utilisation	Y	Y	Y	Y	Y	Y	Y
Brunton	2014	Community engagement	“Given that interventions targeting social determinants of health are necessarily complex (Medical Research Council 2008), this method [QCA] is well-suited to examine the components of effective interventions in maternity and early years interventions.”	Fuzzy	OECD countries (USA, UK, RoI)	Disadvantaged pregnant women and new mothers	Community engagement – orientated to antenatal care, breastfeeding, child illness prevention and parenting	Published analysis (systematic review)	Quant	Intervention	24 of 29 studies (a sub-set of the 131 trials included in the braoder review of studies)	Effectiveness of the intervention.	N	Y	Y	Y	Y	Y	Y
Burchett	2018	Nutrition/ obesity	QCA method used to attend to “the inherent variance in intervention content, context and outcomes […] to explore the critical features of WMPs [weight management programs] for children to understand the mechanisms through which interventions have the impact that they do.”	Crisp	UK	Children and Young People	Lifestyle weight management interventions	Published analysis	Quant	Intervention	15 (most/least effective) of 30 interventions	Intervention effectiveness	N	Y	N	N	N	Y	N
Chippone	2018	Nutrition/ obesity & PA	“to determine necessary and sufficient technical assistance conditions supporting NAP SACC [Nutrition and Physical Activity Self-Assessment in Child Care] outcomes.”; “to account for equifinality”	Fuzzy	USA	Children and Young People	Technical assistance	Primary data (generated for study)	Quant	Intervention (‘ECE programs’)	15 highest performing programs and 15 lowest performing programs (from 10 collaboratives, comprising 84 early care and education programs)	Effectiveness of technical assistance	Y	Y	N	N	Y	N	N
Fernald	2018	Chronic condition management	“to identify conditions/sets important for successful implementation. Qualitative Comparative Analysis (QCA) maintains complexity in the analytical process and allows for multiple pathways to achieve the outcome”	Fuzzy	USA	Primary care patients	Community-Created Self-Management Support (SMS) Tools in Primary Care Practices	Primary data (generated for study)	Qual	Organisation (primary care practice)	16 practices of 16 enrolled in study	Routine SMS implementation in primary care setting	Y	Y	Y	N	Y	Y	Y
Ford	2005	General population health status	“allows for the exploration of the necessity, sufficiency and interactions among the three core public health functions and relates them to the outcome of interest—health impact assessments.”	Crisp	USA	Whole population	Assessment, assurance and policy development functions (Adherence to IoM’s recommendations)	Secondary data	Quant	Region/ subnational area: State	41 of 50 states	Population health improvement 1990–2000 (composite measure based on United Health Group’s ratings) above or below average	N	Y	N	N	Y	Y	Y
Glatman-Freedman	2010	Vaccine adoption and implementation	“to examine the alternative combinations of factors that are conducive to the success or failure of new vaccine introduction”	Crisp	African countries eligible for GAVI funding	Organisations	GAVI funding	Secondary data	Quant	Country	35 (all eligible countries)	Country is in one of 3 groups: with both Hep B and Hib vaccines introduced; just hep b introduced; or neither introduced	N	Y	N	N	N	Y	Y
Harris	2019	Chronic condition management	“to identify the combination of intervention components and processes that are aligned with successful intervention implementation”	Fuzzy	Any (“Most studies were conducted in North America in socially disadvantaged populations.”)	Children and Young People	Asthma management interventions (“if their purpose was to help children improve management of their asthma by increasing knowledge, enhancing skills, or changing behaviour.”)	Published analysis (systematic review)	Qual and Quant	Intervention	33 of 55 studies	Implementation of intervention successful	Y	Y	Y	Y	Y	Y	Y
Harting	2019	Programme implementation	“Our theoretical framework indicates that it is the combination of conditions that is important for network performance, rather than the influence of conditions separately. Therefore, we performed a fuzzy set qualitative comparative analysis (fsQCA)”	Fuzzy	Netherlands, municipalities	Policy actors	Multi-sectoral network Gezonde Slagkracht (Decisive Action for Health) program which provided resources for multisectoral networks for action on overweight, alcohol and drug abuse and/or smoking	Primary data (generated for study)	Quant	Public Health Policy Networks (in municipalities or collaborations of municipalities)	29 of the 34 Dutch public health policy networks in the programme	Network performance - indicated by implementation of more than 3 non-educational PH strategies	N	Y	Y	Y	Y	Y	Y
Hartman-Boyce	2018	Nutrition /obesity	“to identify combinations of intervention components associatedwith statistically significant changes (*P* < 0.05) in the desired direction for at least one of the foods targeted by the intervention […] to augment the narrative review.”	Crisp	Any	Food store consumers	Food purchasing interventions	Published analyses (systematic review)	Quant	Intervention	89 interventions, reported in 35 studies	Sucessful implementation of intervention (“statistically significant changes in the desired direction for ≥1 of the foods targeted by the intervention”)	N	Y	N	N	N	Y	Y
Kahwati	2011	Nutrition /obesity & PA	“to identify facility conditions or combinations of conditions associated with larger 6-month patient weight-loss outcomes. QCA is a method that allows for systematic cross-case comparison to better understand causal complexity.”	Crisp	USA	Adults (Veterans exposed to the MOVE! Weight Management Program)	MOVE! Weight Management Program for Veterans	Primay data and secondary data	Qual and Quant	Organisation - health facilities	Twenty-two facilities of 239 facilities eligible for selection (“Eleven sites with larger outcomes and 11 sites with smaller outcomes”)	Intervention effectiveness (“larger patient weight-loss outcomes”)	Y	Y	Y	N	Y	Y	Y
Kane	2017	Programme implementation	“To determine which combination of elements of capacity is most prevalent among the awardees that successfully implemented objectives”; “Programs such as CCPW [Communities Putting Prevention to Work] invove multiple components that may work together and different program models can lead to success. This method accommodates the complexity associated with evaluating such programs.”	Crisp	USA	Organisations	Communities Putting Prevention to Work (CPPW) program	Primary data (generated for study)	Qual	Organisation - awardee (“In most cases, the lead organization for an awardee was a city or county health department, although community-based organizations served as leads for a few awardees”)	22 of 50 community awardees	Intervention implementation effectivesness (“completion of approximately 60% of work plan objectives”)	Y	Y	Y	N	Y	Y	Y
Kien	2018	PA	“We chose QCA as the method of analysis, as we investigated a small number of cases and aimed to answer a question related to the combinations of conditions and not related to the identification of the independent influence of a variable. Furthermore, we were interested, in whether different combinations of causal conditions could lead to the same outcome.”	Fuzzy	Austria	Children and Young People	School based health promotion programme: “Classes in Motion”	Primary data (generated for study)	Qual and Quant	Classes	24 classes out of 26 classes that received the intervention	Percent of children showing an improvement in emotional and social school experience.	Y	Y	Y	Y	Y	Y	Y
Lubold	2017	Breastfeeding	“to examine the combinations of conditions leading to both high and low national breastfeeding initiation”	Fuzzy	OECD countries	Mothers	Baby-Friendly Hospital Initiative (BFHI)	Secondary data	Quant	Country	18 countries of 34 OECD countries	Percent of breastfeeding initiation	N	Y	N	Y	Y	Y	Y
Lucidarme	2016	Programme implementation	QCA as most appropriate method because “we have a combination of a relatively large number of determinants and a small number of cases.”; “a large amount of in-depth qualitative data was collected and CCMs [Configurational comparative methods] are able to deal with such large amounts of qualitative data.”	Crisp	Flanders, Belgium	Organisations	Local health promotion networks (LHP)	Primary data (generated for study) Secondary data	Qual and Quant	Network	13 of 13 LHPs	Composite score of effectiveness of network from 5 measures at Community level (Awareness and change in awareness of ‘10,000 steps’ programme) and Network level (measures of participation, and actions at municipal and regional level)	Y	Y	Y	Y	Y	Y	Y
McGowan	2019	Mental health & Community Engagement	“QCA is therefore of potential use in the evaluation of the effectiveness of complex public health interventions as applied to small populations”	Crisp	UK, ‘Big Local’ (BL) areas (disadvantaged areas in UK)	Adult participants in BL partnerships (residents, workers, volunteers in locality)	A community-led empowerent initiative	Primary data (generated for study)	Quant	Person /individual	48 participants of 65 participating in a Wave 2 survey	Improvement in mental health	N	Y	Y	N	Y	N	N
Melendez-Torres	2018	Nutrition /obesity & PA	“we aimed to understand why some interventions appeared to work better than others, or, whether specific combinations of WMP [weight management program] features were associated with greater intervention effectiveness. [...] QCA was particularly suitable [...] as it facilitates the identification of configurations of various intervention and other contextual features that are (or are not) present when the intervention has been evaluated and found successful (or not) in obtaining a desired outcome”	Fuzzy	Any (but notes in original review that over half of included studies were from US)	Overweight or obese adults	Weight management programs	Published analysis	Quant	Intervention	20 (10 most effective; 10 least effective) from 40 intervention arms within 30 trials	Pathways to high (and low) intervention effectivess	N	Y	Y	Y	Y	Y	Y
Parrott	2018	Nutrition/obesity	“QCA is much more suited to answering the question: For whom and under what conditions does the intervention make a difference”	Crisp	Any	Children and Young People	Pediatric weight management (PWM) interventions	Published analysis (systematic review)	Quant	Intervention component	209 separate treatment arms from 99 controlled trials, were included in this analysis.	Positive weight status outcomes	N	Y	Y	N	Y	Y	Y
Roberts	2018	Vaccine adoption and implementation	“Qualitative comparative analysis (QCA) is a formalized qualitative analytic approach that can be leveraged to determine which sets of state policies may be necessary or sufficient for high state-level HPV vaccination uptake. States have enacted multiple, often overlapping policies that may influence HPV vaccine uptake; QCA is well suited for characterizing these complex relationships.”	Fuzzy	USA	Children and Young People	State policies	Secondary data	Quant	Region/subnational area	51 (of 50 States + Washington DC)	Higher levels of HPV vaccine uptake in adolescent boys and girls	N	Y	Y	N	Y	Y	Y
Thomas	2014	Community engagement & Breastfeeding	“the context for this paper: a need to identify important components of interventions when making commissioning decisions, but a lack of established methods of synthesis which enable such investigations. We therefore examine an analytical technique, [QCA] which has been designed to overcome some of the limitations outlined above.”	Fuzzy	OECD countries	Expectant and new mothers	Community engagement programmes directed toward expectant and new mothers to promote breastfeeding.	Published analysis (systematic review)	Quant	Intervention	12 studies as a subset of a review that included 319 studies	Intervention effectiveness (“membership in the set of highly effective interventions”)	N	Y	Y	Y	Y	Y	Y
Warren	2013, 2014	Chronic condition management	To capture complexity (particularly on how policy interventions work across heterogeneous contexts) (2013); “QCA addresses multiple causation” (2014)	Crisp	UK - North-East England	Adult working age recipients of incapacity benefit (IB)	Case management to help benefit recipients return to work	Primary data	Quant	Individual	131 participants receiving the intervention	Self-reported health (EQ. 5-S score) improves/does not improve relative to UK population	Y	Y	N	Y	Y	Y	Y

### Quality assessment

There are no reporting guidelines for QCA studies in public health, but there are a number of discussions of best practice in the methodological literature [25, 26, 33, 34]. These discussions suggest several criteria for strengthening QCA methods that we used as indicators of methodological and/or reporting quality: evidence of familiarity of cases; justification for selection of cases; discussion and justification of set membership score calibration; reporting of truth tables; reporting and justification of solution formula; and reporting of consistency and coverage measures. For studies using csQCA, and claiming an explanatory analysis, we additionally identified whether the number of cases was sufficient for the number of conditions included in the model, using a pragmatic cut-off in line with Marx & Dusa’s guideline thresholds, which indicate how many cases are sufficient for given numbers of conditions to reject a 10% probability that models could be generated with random data [26].

## Results

### Overview of scope of QCA research in public health

Twenty-seven papers reporting 26 studies were included in the review (Table 1). The earliest was published in 2005, and 17 were published after 2015. The majority (*n* = 19) were published in public health/health promotion journals, with the remainder published in other health science (*n* = 3) or in social science/management journals (*n* = 4). The public health domain(s) addressed by each study were broadly coded by the main area of focus. They included nutrition/obesity (*n* = 8); physical activity (PA) (n = 4); health inequalities (*n* = 3); mental health (*n* = 2); community engagement (*n *= 3); chronic condition management (*n *= 3); vaccine adoption or implementation (n = 2); programme implementation (*n *= 3); breastfeeding (*n *= 2); or general population health (*n* = 1). The majority (*n* = 24) of studies were conducted solely or predominantly in high-income countries (systematic reviews in general searched global sources, but commented that the overwhelming majority of studies were from high-income countries). Country settings included: any (*n* = 6); OECD countries (*n* = 3); USA (*n* = 6); UK (*n* = 6) and one each from Nepal, Austria, Belgium, Netherlands and Africa. These largely reflected the first author’s country affiliations in the UK (*n* = 13); USA (*n* = 9); and one each from South Africa, Austria, Belgium, and the Netherlands. All three studies primarily addressing health inequalities [35–37] were from the UK.

Eight of the interventions evaluated were individual-level behaviour change interventions (e.g. weight management interventions, case management, self-management for chronic conditions); eight evaluated policy/funding interventions; five explored settings-based health promotion/behaviour change interventions (e.g. schools-based physical activity intervention, store-based food choice interventions); three evaluated community empowerment/engagement interventions, and two studies evaluated networks and their impact on health outcomes.

### Methods and data sets used

Fifteen studies used crisp sets (csQCA), 11 used fuzzy sets (fsQCA). No study used mvQCA. Eleven studies included additional analyses of the datasets drawn on for the QCA, including six that used qualitative approaches (narrative synthesis, case comparisons), typically to identify cases or conditions for populating the QCA; and four reporting additional statistical analyses (meta-regression, linear regression) to either identify differences overall between cases prior to conducting a QCA (e.g. [38]) or to explore correlations in more detail (e.g. [39]). One study used an additional Boolean configurational technique to reduce the number of conditions in the QCA analysis [40]. No studies reported aiming to compare the findings from the QCA with those from other techniques for evaluating the uptake or effectiveness of interventions, although some [41, 42] were explicitly using the study to showcase the possibilities of QCA compared with other approaches in general. Twelve studies drew on primary data collected specifically for the study, with five of those additionally drawing on secondary data sets; five drew only on secondary data sets, and nine used data from systematic reviews of published research. Seven studies drew primarily on qualitative data, generally derived from interviews or observations.

Many studies were undertaken in the context of one or more trials, which provided evidence of effect. Within single trials, this was generally for a process evaluation, with cases being trial sites. Fernald et al’s study, for instance, was in the context of a trial of a programme to support primary care teams in identifying and implementing self-management support tools for their patients, which measured patient and health care provider level outcomes [43]. The QCA reported here used qualitative data from the trial to identify a set of necessary conditions for health care provider practices to implement the tools successfully. In studies drawing on data from systematic reviews, cases were always at the level of intervention or intervention component, with data included from multiple trials. Harris et al., for instance, undertook a mixed-methods systematic review of school-based self-management interventions for asthma, using meta-analysis methods to identify effective interventions and QCA methods to identify which intervention features were aligned with success [44].

### Cases

The largest number of studies (*n* = 10), including all the systematic reviews, analysed cases at the level of the intervention, or a component of the intervention; seven analysed organisational level cases (e.g. school class, network, primary care practice); five analysed sub-national region level cases (e.g. state, local authority area), and two each analysed country or individual level cases. Sample sizes ranged from 10 to 131, with no study having small N (< 10) sample sizes, four having large N (> 50) sample sizes, and the majority (22) being medium N studies (in the range 10–50).

### Rationale for using QCA

Most papers reported a rationale for using QCA that mentioned ‘complexity’ or ‘context’, including: noting that QCA is appropriate for addressing causal complexity or multiple pathways to outcome [37, 43, 45–51]; noting the appropriateness of the method for providing evidence on how context impacts on interventions [41, 50]; or the need for a method that addressed causal asymmetry [52]. Three stated that the QCA was an ‘exploratory’ analysis [53–55]. In addition to the empirical aims, several papers (e.g. [42, 48]) sought to demonstrate the utility of QCA, or to develop QCA methods for health research (e.g. [47]).

### Reported strengths and weaknesses of approach

There was a general agreement about the strengths of QCA. Specifically, that it was a useful tool to address complex causality, providing a systematic approach to understand the mechanisms at work in implementation across contexts [38, 39, 43, 45–47, 55–57], particularly as they relate to (in) effective intervention implementation [44, 51] and the evaluation of interventions [58], or “where it is not possible to identify linearity between variables of interest and outcomes” [49]. Authors highlighted the strengths of QCA as providing possibilities for examining complex policy problems [37, 59]; for testing existing as well as new theory [52]; and for identifying aspects of interventions which had not been previously perceived as critical [41] or which may have been missed when drawing on statistical methods that use, for instance, linear additive models [42]. The strengths of QCA in terms of providing useful evidence for policy were flagged in a number of studies, particularly where the causal recipes suggested that conventional assumptions about effectiveness were not confirmed. Blackman et al., for instance, in a series of studies exploring why unequal health outcomes had narrowed in some areas of the UK and not others, identified poorer outcomes in settings with ‘better’ contracting [35–37]; Harting found, contrary to theoretical assumptions about the necessary conditions for successful implementation of public health interventions, that a multisectoral network was not a necessary condition [30].

Weaknesses reported included the limitations of QCA in general for addressing complexity, as well as specific limitations with either the csQCA or the fsQCA methods employed. One general concern discussed across a number of studies was the problem of limited empirical diversity, which resulted in: limitations in the possible number of conditions included in each study, particularly with small N studies [58]; missing data on important conditions [43]; or limited reported diversity (where, for instance, data were drawn from systematic reviews, reflecting publication biases which limit reporting of ineffective interventions) [41]. Reported methodological limitations in small and intermediate N studies included concerns about the potential that case selection could bias findings [37].

In terms of potential for addressing causal complexity, the limitations of QCA for identifying unintended consequences, tipping points, and/or feedback loops in complex adaptive systems were noted [60], as were the potential limitations (especially in csQCA studies) of reducing complex conditions, drawn from detailed qualitative understanding, to binary conditions [35]. The impossibility of doing this was a rationale for using fsQCA in one study [57], where detailed knowledge of conditions is needed to make theoretically justified calibration decisions. However, others [47] make the case that csQCA provides more appropriate findings for policy: dichotomisation forces a focus on meaningful distinctions, including those related to decisions that practitioners/policy makers can action. There is, then, a potential trade-off in providing ‘interpretable results’, but ones which preclude potential for utilising more detailed information [45]. That QCA does not deal with probabilistic causation was noted [47].

### Quality of published studies

Assessment of ‘familiarity with cases’ was made subjectively on the basis of study authors’ reports of their knowledge of the settings (empirical or theoretical) and the descriptions they provided in the published paper: overall, 14 were judged as sufficient, and 12 less than sufficient. Studies which included primary data were more likely to be judged as demonstrating familiarity (*n* = 10) than those drawing on secondary sources or systematic reviews, of which only two were judged as demonstrating familiarity. All studies justified how the selection of cases had been made; for those not using the full available population of cases, this was in general (appropriately) done theoretically: following previous research [52]; purposively to include a range of positive and negative outcomes [41]; or to include a diversity of cases [58]. In identifying conditions leading to effective/not effective interventions, one purposive strategy was to include a specified percentage or number of the most effective and least effective interventions (e.g. [36, 40, 51, 52]). Discussion of calibration of set membership scores was judged adequate in 15 cases, and inadequate in 11; 10 reported raw data matrices in the paper or supplementary material; 21 reported truth tables in the paper or supplementary material. The majority (*n* = 21) reported at least some detail on the coverage (the number of cases with a particular configuration) and consistency (the percentage of similar causal configurations which result in the same outcome). The majority (*n* = 21) included truth tables (or explicitly provided details of how to obtain them); fewer (*n* = 10) included raw data. Only five studies met all six of these quality criteria (evidence of familiarity with cases, justification of case selection, discussion of calibration, reporting truth tables, reporting raw data matrices, reporting coverage and consistency); a further six met at least five of them.

Of the csQCA studies which were not reporting an exploratory analysis, four appeared to have insufficient cases for the large number of conditions entered into at least one of the models reported, with a consequent risk to the validity of the QCA models [26].

## Discussion

QCA has been widely used in public health research over the last decade to advance understanding of causal inference in complex systems. In this review of published evidence to date, we have identified studies using QCA to examine the configurations of conditions that lead to particular outcomes across contexts. As noted by most study authors, QCA methods have promised advantages over probabilistic statistical techniques for examining causation where systems and/or interventions are complex, providing public health researchers with a method to test the multiple pathways (configurations of conditions), and necessary and sufficient conditions that lead to desired health outcomes.

The origins of QCA approaches are in comparative policy studies. Rihoux et al’s review of peer-reviewed journal articles using QCA methods published up to 2011 found the majority of published examples were from political science and sociology, with fewer than 5% of the 313 studies they identified coming from health sciences [61]. They also reported few examples of the method being used in policy evaluation and implementation studies [62]. In the decade since their review of the field [61], there has been an emerging body of evaluative work in health: we identified 26 studies in the field of public health alone, with the majority published in public health journals. Across these studies, QCA has been used for evaluative questions in a range of settings and public health domains to identify the conditions under which interventions are implemented and/or have evidence of effect for improving population health. All studies included a series of cases that included some with and some without the outcome of interest (such as behaviour change, successful programme implementation, or good vaccination uptake). The dominance of high-income countries in both intervention settings and author affiliations is disappointing, but reflects the disproportionate location of public health research in the global north more generally [63].

The largest single group of studies included were systematic reviews, using QCA to compare interventions (or intervention components) to identify successful (and non-successful) configurations of conditions across contexts. Here, the value of QCA lies in its potential for synthesis with quantitative meta-synthesis methods to identify the particular conditions or contexts in which interventions or components are effective. As Parrott et al. note, for instance, their meta-analysis could identify probabilistic effects of weight management programmes, and the QCA analysis enabled them to address the “role that the context of the [paediatric weight management] intervention has in influencing how, when, and for whom an intervention mix will be successful” [50]. However, using QCA to identify configurations of conditions that lead to effective or non- effective interventions across particular areas of population health is an application that does move away in some significant respects from the origins of the method. First, researchers drawing on evidence from systematic reviews for their data are reliant largely on published evidence for information on conditions (such as the organisational contexts in which interventions were implemented, or the types of behaviour change theory utilised). Although guidance for describing interventions [64] advises key aspects of context are included in reports, this may not include data on the full range of conditions that might be causally important, and review research teams may have limited knowledge of these ‘cases’ themselves. Second, less successful interventions are less likely to be published, potentially limiting the diversity of cases, particularly of cases with unsuccessful outcomes. A strength of QCA is the separate analysis of conditions leading to positive and negative outcomes: this is precluded where there is insufficient evidence on negative outcomes [50]. Third, when including a range of types of intervention, it can be unclear whether the cases included are truly comparable. A QCA study requires a high degree of theoretical and pragmatic case knowledge on the part of the researcher to calibrate conditions to qualitative anchors: it is reliant on deep understanding of complex contexts, and a familiarity with how conditions interact within and across contexts. Perhaps surprising is that only seven of the studies included here clearly drew on qualitative data, given that QCA is primarily seen as a method that requires thick, detailed knowledge of cases, particularly when the aim is to understand complex causation [8]. Whilst research teams conducting QCA in the context of systematic reviews may have detailed understanding in general of interventions within their spheres of expertise, they are unlikely to have this for the whole range of cases, particularly where a diverse set of contexts (countries, organisational settings) are included. Making a theoretical case for the valid comparability of such a case series is crucial. There may, then, be limitations in the portability of QCA methods for conducting studies entirely reliant on data from published evidence.

QCA was developed for small and medium N series of cases, and (as in the field more broadly, [61]), the samples in our studies predominantly had between 10 and 50 cases. However, there is increasing interest in the method as an alternative or complementary technique to regression-oriented statistical methods for larger samples [65], such as from surveys, where detailed knowledge of cases is likely to be replaced by theoretical knowledge of relationships between conditions (see [23]). The two larger N (> 100 cases) studies in our sample were an individual level analysis of survey data [46, 47] and an analysis of intervention arms from a systematic review [50]. Larger sample sizes allow more conditions to be included in the analysis [23, 26], although for evaluative research, where the aim is developing a causal explanation, rather than simply exploring patterns, there remains a limit to the number of conditions that can be included. As the number of conditions included increases, so too does the number of possible configurations, increasing the chance of unique combinations and of generating spurious solutions with a high level of consistency. As a rule of thumb, once the number of conditions exceeds 6–8 (with up to 50 cases) or 10 (for larger samples), the credibility of solutions may be severely compromised [23].

### Strengths and weaknesses of the study

A systematic review has the potential advantages of transparency and rigour and, if not exhaustive, our search is likely to be representative of the body of research using QCA for evaluative public health research up to 2020. However, a limitation is the inevitable difficulty in operationalising a ‘public health’ intervention. Exclusions on scope are not straightforward, given that most social, environmental and political conditions impact on public health, and arguably a greater range of policy and social interventions (such as fiscal or trade policies) that have been the subject of QCA analyses could have been included, or a greater range of more clinical interventions. However, to enable a manageable number of papers to review, and restrict our focus to those papers that were most directly applicable to (and likely to be read by) those in public health policy and practice, we operationalised ‘public health interventions’ as those which were likely to be directly impacting on population health outcomes, or on behaviours (such as increased physical activity) where there was good evidence for causal relationships with public health outcomes, and where the primary research question of the study examined the conditions leading to those outcomes. This review has, of necessity, therefore excluded a considerable body of evidence likely to be useful for public health practice in terms of planning interventions, such as studies on how to better target smoking cessation [66] or foster social networks [67] where the primary research question was on conditions leading to these outcomes, rather than on conditions for outcomes of specific interventions. Similarly, there are growing number of descriptive epidemiological studies using QCA to explore factors predicting outcomes across such diverse areas as lupus and quality of life [68]; length of hospital stay [69]; constellations of factors predicting injury [70]; or the role of austerity, crisis and recession in predicting public health outcomes [71]. Whilst there is undoubtedly useful information to be derived from studying the conditions that lead to particular public health problems, these studies were not directly evaluating interventions, so they were also excluded.

Restricting our search to publications in English and to peer reviewed publications may have missed bodies of work from many regions, and has excluded research from non-governmental organisations using QCA methods in evaluation. As this is a rapidly evolving field, with relatively recent uptake in public health (all our included studies were after 2005), our studies may not reflect the most recent advances in the area.

### Implications for conducting and reporting QCA studies

This systematic review has reviewed studies that deployed an emergent methodology, which has no reporting guidelines and has had, to date, a relatively low level of awareness among many potential evidence users in public health. For this reason, many of the studies reviewed were relatively detailed on the methods used, and the rationale for utilising QCA.

We did not assess quality directly, but used indicators of good practice discussed in QCA methodological literature, largely written for policy studies scholars, and often post-dating the publication dates of studies included in this review. It is also worth noting that, given the relatively recent development of QCA methods, methodological debate is still thriving on issues such as the reliability of causal inferences [72], alongside more general critiques of the usefulness of the method for policy decisions (see, for instance, [73]). The authors of studies included in this review also commented directly on methodological development: for instance, Thomas et al. suggests that QCA may benefit from methods development for sensitivity analyses around calibration decisions [42].

However, we selected quality criteria that, we argue, are relevant for public health research> Justifying the selection of cases, discussing and justifying the calibration of set membership, making data sets available, and reporting truth tables, consistency and coverage are all good practice in line with the usual requirements of transparency and credibility in methods. When QCA studies aim to provide explanation of outcomes (rather than exploring configurations), it is also vital that they are reported in ways that enhance the credibility of claims made, including justifying the number of conditions included relative to cases. Few of the studies published to date met all these criteria, at least in the papers included here (although additional material may have been provided in other publications). To improve the future discoverability and uptake up of QCA methods in public health, and to strengthen the credibility of findings from these methods, we therefore suggest the following criteria should be considered by authors and reviewers for reporting QCA studies which aim to provide causal evidence about the configurations of conditions that lead to implementation or outcomes:

1. The paper title and abstract state the QCA design;
2. The sampling unit for the ‘case’ is clearly defined (e.g.: patient, specified geographical population, ward, hospital, network, policy, country);
3. The population from which the cases have been selected is defined (e.g.: all patients in a country with X condition, districts in X country, tertiary hospitals, all hospitals in X country, all health promotion networks in X province, European policies on smoking in outdoor places, OECD countries);
4. The rationale for selection of cases from the population is justified (e.g.: whole population, random selection, purposive sample);
5. There are sufficient cases to provide credible coverage across the number of conditions included in the model, and the rationale for the number of conditions included is stated;
6. Cases are comparable;
7. There is a clear justification for how choices of relevant conditions (or ‘aspects of context’) have been made;
8. There is sufficient transparency for replicability: in line with open science expectations, datasets should be available where possible; truth tables should be reported in publications, and reports of coverage and consistency provided.

### Implications for future research

In reviewing methods for evaluating natural experiments, Craig et al. focus on statistical techniques for enhancing causal inference, noting only that what they call ‘qualitative’ techniques (the cited references for these are all QCA studies) require “further studies … to establish their validity and usefulness” [2]. The studies included in this review have demonstrated that QCA is a feasible method when there are sufficient (comparable) cases for identifying configurations of conditions under which interventions are effective (or not), or are implemented (or not). Given ongoing concerns in public health about how best to evaluate interventions across complex contexts and systems, this is promising. This review has also demonstrated the value of adding QCA methods to the tool box of techniques for evaluating interventions such as public policies, health promotion programmes, and organisational changes - whether they are implemented in a randomised way or not. Many of the studies in this review have clearly generated useful evidence: whether this evidence has had more or less impact, in terms of influencing practice and policy, or is more valid, than evidence generated by other methods is not known. Validating the findings of a QCA study is perhaps as challenging as validating the findings from any other design, given the absence of any gold standard comparators. Comparisons of the findings of QCA with those from other methods are also typically constrained by the rather different research questions asked, and the different purposes of the analysis. In our review, QCA were typically used alongside other methods to address different questions, rather than to compare methods. However, as the field develops, follow up studies, which evaluate outcomes of interventions designed in line with conditions identified as causal in prior QCAs, might be useful for contributing to validation.

This review was limited to public health evaluation research: other domains that would be useful to map include health systems/services interventions and studies used to design or target interventions. There is also an opportunity to broaden the scope of the field, particularly for addressing some of the more intractable challenges for public health research. Given the limitations in the evidence base on what works to address inequalities in health, for instance [74], QCA has potential here, to help identify the conditions under which interventions do or do not exacerbate unequal outcomes, or the conditions that lead to differential uptake or impacts across sub-population groups. It is perhaps surprising that relatively few of the studies in this review included cases at the level of country or region, the traditional level for QCA studies. There may be scope for developing international comparisons for public health policy, and using QCA methods at the case level (nation, sub-national region) of classic policy studies in the field. In the light of debate around COVID-19 pandemic response effectiveness, comparative studies across jurisdictions might shed light on issues such as differential population responses to vaccine uptake or mask use, for example, and these might in turn be considered as conditions in causal configurations leading to differential morbidity or mortality outcomes.

#### When should be QCA be considered?

Public health evaluations typically assess the efficacy, effectiveness or cost-effectiveness of interventions and the processes and mechanisms through which they effect change. There is no perfect evaluation design for achieving these aims. As in other fields, the choice of design will in part depend on the availability of counterfactuals, the extent to which the investigator can control the intervention, and the range of potential cases and contexts [75], as well as political considerations, such as the credibility of the approach with key stakeholders [76]. There are inevitably ‘horses for courses’ [77]. The evidence from this review suggests that QCA evaluation approaches are feasible when there is a sufficient number of comparable cases with and without the outcome of interest, and when the investigators have, or can generate, sufficiently in-depth understanding of those cases to make sense of connections between conditions, and to make credible decisions about the calibration of set membership. QCA may be particularly relevant for understanding multiple causation (that is, where different configurations might lead to the same outcome), and for understanding the conditions associated with both lack of effect *and* effect. As a stand-alone approach, QCA might be particularly valuable for national and regional comparative studies of the impact of policies on public health outcomes. Alongside cluster randomised trials of interventions, or alongside systematic reviews, QCA approaches are especially useful for identifying core combinations of causal conditions for success and lack of success in implementation and outcome.

## Conclusions

QCA is a relatively new approach for public health research, with promise for contributing to much-needed methodological development for addressing causation in complex systems. This review has demonstrated the large range of evaluation questions that have been addressed to date using QCA, including contributions to process evaluations of trials and for exploring the conditions leading to effectiveness (or not) in systematic reviews of interventions. There is potential for QCA to be more widely used in evaluative research, to identify the conditions under which interventions across contexts are implemented or not, and the configurations of conditions associated with effect or lack of evidence of effect. However, QCA will not be appropriate for all evaluations, and cannot be the only answer to addressing complex causality. For explanatory questions, the approach is most appropriate when there is a series of enough comparable cases with and without the outcome of interest, and where the researchers have detailed understanding of those cases, and conditions. To improve the credibility of findings from QCA for public health evidence users, we recommend that studies are reported with the usual attention to methodological transparency and data availability, with key details that allow readers to judge the credibility of causal configurations reported. If the use of QCA continues to expand, it may be useful to develop more comprehensive consensus guidelines for conduct and reporting.

## Supplementary Information

**Additional file 1.** Example search strategy.

## Data Availability

Full search strategies and extraction forms are available by request from the first author.

## Abbreviations

COMPASSS: Comparative Methods for Systematic Cross-Case Analysis
csQCA: crisp set QCA
fsQCA: fuzzy set QCA
mvQCA: multi-value QCA
MRC: Medical Research Council
QCA: Qualitative Comparative Analysis
RCT: randomised control trial
PA: Physical Activity
