# Supplementary material for: The use of Qualitative Comparative Analysis (QCA) to address causality in complex systems: a systematic review of research on public health interventions
Source: BMC Public Health. 2021 May 7;21:877. doi: 10.1186/s12889-021-10926-2 (PMC8103124; doi:10.1186/s12889-021-10926-2)
Supplement: Supplementary file 1 — Additional file 1. Example search strategy. [file 12889_2021_10926_MOESM1_ESM.docx]

**The use of Qualitative Comparative Analysis (QCA) to address causality in complex systems: a systematic review of research on public health interventions**

Benjamin Hanckel^1^, Mark Petticrew^2^, James Thomas ^3^, Judith Green ^4*^

1. Institute for Culture and Society, Western Sydney University, Australia b.hanckel@westernsydney.edu.au
2. Department of Public Health Environment and Society, LSHTM, UK

Mark.petticrew@lshtm.ac.uk

1. UCL Institute of Education, University College London, UK

James.thomas@ucl.ac.uk

1. Wellcome Centre for Cultures & Environments of Health, University of Exeter, UK

[j.m.green@exeter.ac.uk](mailto:j.m.green@exeter.ac.uk)

*corresponding author

**Appendix 1. Example search strategy**

The search strategy below was used in the Web of Science database. A translated version was used in the other databases.

**Web of Science** (all databases selected; English only).

No. of records: 916

| **Set** | **Searches** |
| --- | --- |
| **1** | Qualitative Comparative Analysis AND Health (179 articles) |
| **2** | QCA AND Health (128) |
| **3** | Qualitative Comparative Analysis AND Public Health (47 articles) |
| **4** | QCA AND Public Health (42) |
| **5** | Qualitative Comparative Analysis AND intervention (71 articles) |
| **6** | QCA AND Intervention (446) |
| **7** | Qualitative Comparative Analysis AND wellbeing (2 articles) |
| **8** | QCA AND Wellbeing (1) |
